# Effects of beta-blockers use on mortality of patients with acute respiratory distress syndrome: a retrospective cohort study

**DOI:** 10.3389/fphys.2024.1332571

**Published:** 2024-01-19

**Authors:** Yukang Dong, Run Sun, Jiangquan Fu, Rui Huang, Huan Yao, Jingni Wang, Ying Wang, Feng Shen

**Affiliations:** ^1^ Department of Intensive Care Unit, Guizhou Medical University Affiliated Hospital, Guiyang, China; ^2^ Department of Emergency, Guizhou Provincial People’s Hospital, Guiyang, China; ^3^ Department of Emergency Intensive Care Unit, Guizhou Medical University Affiliated Hospital, Guiyang, China; ^4^ Xiangya School of Nursing, Central South University, Changsha, Hunan, China; ^5^ Nursing Department, Guizhou Provincial People’s Hospital, Guiyang, Guizhou, China

**Keywords:** beta-blockers, acute respiratory distress syndrome, retrospective cohort study, mortality, MIMIC IV databases

## Abstract

**Introduction:** Acute respiratory distress syndrome (ARDS) remains a challenging disease with limited prevention and treatment options. The usage of beta-blockers may have potential benefits in different critical illnesses. This study aimed to investigate the correlation between beta-blocker therapy and mortality in patients with ARDS.

**Materials and methods:** This retrospective cohort study utilized data from the Medical Information Mart for Intensive Care (MIMIC) IV database and focused on patients diagnosed with ARDS. The primary outcome of the study was 30-day mortality. To account for confounding factors, a multivariable analysis was performed. Propensity score matching (PSM) was carried out on a 1:1 ratio. Robust assessments were conducted using inverse probability weighting (IPTW), standardized mortality ratio weighting (SMRW), pairwise algorithms (PA), and overlap weights (OW).

**Results:** A total of 1,104 patients with ARDS were included in the study. Univariate and multivariate Cox regression analyses found that the 30-day mortality for 489 patients (23.7%) who received beta-blockers was significantly lower than the mortality rate of 615 patients (35.9%) who did not receive beta-blockers. After adjusting for potential confounders through PSM and propensity score, as well as utilizing IPTW, SMRW, PA, and OW, the results remained robust, with the hazard ratios (HR) ranging from 0.42 to 0.58 and all *p*-values < 0.001. Evaluation of the E-values indicated the robustness of the results even in the presence of unmeasured confounding.

**Conclusion:** The findings suggest a potential association between beta-blocker usage and reduced mortality in critically ill patients with ARDS. However, further validation of this observation is needed through randomized controlled trials.

## 1 Introduction

Intensive care units have been grappling with acute respiratory distress syndrome (ARDS) for over half a century, posing an ongoing formidable challenge (Pham and Rubenfeld, 2017). Globally, approximately three million patients are affected by ARDS annually, accounting for approximately 10% of ICU admissions (Rubenfeld et al., 2005; Bellani et al., 2016; Fan et al., 2018). Despite various therapeutic attempts, mortality rates related to ARDS have not decreased since the late nineties (Phua et al., 2009; Herridge et al., 2011). ARDS differs from simple pulmonary diseases as it is a clinical syndrome with diverse potential etiologies and pathophysiological characteristics. Its development and occurrence are not necessarily linked to primary lung injury but may result from an unregulated host/organ response (HOUR), commonly referred to as the “second hit” (Pan et al., 2021; Huang et al., 2023). Effective management of acute respiratory distress syndrome entails evaluating the efficacy of therapeutic interventions and preventing secondary lung injury caused by this “second hit.”

The unregulated host/organ response in critical illness is characterized by increased sympathetic drive resulting from adrenergic stimulation of catecholamines, which is a fundamental pathological feature (Dünser and Hasibeder, 2009). The lung is particularly susceptible to sympathetic overstimulation, often accompanied by hyperdynamic circulation. In lung injury cases, it is hypothesized that enhanced blood flow through the pulmonary vasculature may contribute to or exacerbate acute lung injury or even ARDS (van der Jagt and Miranda, 2012). The potential role of beta-blockers in antagonizing the adrenergic stimulation of catecholamines has been investigated in various critical conditions, such as sepsis, trauma, cardiac arrest, and burns. Growing evidence suggests that beta-blockers improve hemodynamic and metabolic factors, resulting in reduced burn healing durations (Mohammadi et al., 2009; Ali et al., 2015; Cheema et al., 2020), decreased mortality following traumatic brain injury (Mohseni et al., 2015; Ley et al., 2018), and improved neurological outcomes after cardiac arrest (Driver et al., 2014; Lee et al., 2016). In sepsis, beta-blockers may have a beneficial effect on hemodynamics and cardiac function (Morelli et al., 2016), while potentially reducing mortality (Morelli et al., 2013). Some studies have indicated that beta-blockers could reduce lung endothelial damage in ARDS by regulating pulmonary vascular flow without affecting systemic hemodynamics, suggesting their potential as therapeutic agents for ARDS treatment (Piacentini et al., 2008; Hagiwara et al., 2009). However, there is currently insufficient evidence to support the theory that beta-blocker usage reduces mortality in critically ill patients with ARDS. Therefore, a retrospective cohort analysis was conducted to validate the association between beta-blocker usage and mortality among individuals diagnosed with ARDS.

## 2 Materials and methods

### 2.1 Data source

This study included a cohort of patients diagnosed with ARDS who received treatment with or without beta-blockers. Data was extracted from the Medical Information Mart for Intensive Care-IV (MIMIC-IV) database (version 2.0). The MIMIC-IV database comprises clinical information from patients admitted to the Beth Israel Deaconess Medical Center between 2012 and 2019. To gain access to the relevant data, our team successfully completed the Protecting Human Research Participants test, the National Institutes of Health training course, and their respective exams (certification number: 39719844). As this project did not impact clinical care and all protected health information was deidentified, the Institutional Review Boards (IRB) of the Massachusetts Institute of Technology waived the need for an ethical approval statement and informed consent. The findings of this study were reported following the guidelines for “Strengthening the Reporting of Observational Studies in Epidemiology.”

### 2.2 Study population

Screening was conducted for all individuals in the database to ensure eligibility. The criteria for inclusion were as follows: 1) Patients admitted to the ICU who were diagnosed with ARDS according to the Berlin standard; 2) Patients aged ≥18 years; 3) Patients who received invasive ventilation for a minimum of 24 consecutive hours; and 4) Only the initial ICU admission was considered for analysis (Serpa Neto et al., 2018). The exclusion criteria were: 1) Patients who were exclusively administered beta-blockers outside the ICU setting; 2) Patients with acute or chronic heart failure. The Berlin standard for ARDS diagnosis included the following criteria: acute onset, arterial oxygen partial pressure (PaO_2_)/fraction of inspired oxygen (FiO_2_) <300 mmHg, and positive end-expiratory pressure (PEEP) ≥5 cm H_2_O on the first day of ICU admission, along with bilateral infiltrates evident on chest radiograph and the absence of heart failure. ARDS severity grading based on the PaO_2_/FiO_2_ ratio was categorized as mild (>200 mmHg and ≤300 mmHg), moderate (>100 mmHg and ≤ 200 mmHg), and severe (≤100 mmHg) according to the Berlin Definition.

### 2.3 Covariates

The MIMIC-IV database was accessed using Structured Query Language (SQL) to obtain relevant data for analysis. The collected demographic characteristics such as age, gender, ethnicity, and weight were recorded, along with various clinical scores, including Sequential Organ Failure Assessment (SOFA) score and Simplified Acute Physiology Score II (SAPS II) score. Mean values of vital signs such as mean arterial pressure (MAP), heart rate, temperature, respiratory rate, and Spo_2_, as well as measurements of PH, bicarbonate, arterial oxygen partial pressure (PaO_2_), and arterial carbon dioxide tension (PaCO_2_) were also documented. Additionally, comorbidities, including cerebrovascular disease, chronic obstructive pulmonary disease, liver disease, renal disease, malignancy, and sepsis were recorded. Treatment details such as renal replacement therapy (RRT), vasopressor use (norepinephrine or epinephrine), glucocorticoid administration (hydrocortisone, methylprednisolone, prednisone, or dexamethasone), and use of neuromuscular blocker agents (NMBA) (cisatracurium, vecuronium bromide, or rocuronium) were noted. Furthermore, mean values of parameters related to mechanical ventilation, such as the fraction of inspired oxygen, tidal volume, positive end-expiratory pressure (PEEP), driven pressure, and mechanical power, were recorded on the first day of ICU admission to analyze initial baseline characteristics and laboratory results.

### 2.4 Definitions and outcomes

In this study, beta-blocker therapy was defined as the administration of any form of beta-blockers (oral or intravenous), irrespective of the dosage, during an ICU admission. The primary outcome assessed was the 30-day mortality. Furthermore, secondary outcomes considered included the 60-day mortality, length of stay in both the ICU and hospital, as well as the duration of ventilation.

### 2.5 Statistical analysis

Categorical variables were summarized using frequency and percentages, while continuous variables were described using the median with the interquartile range (IQR) for skewed distributions and the mean with standard deviation (SD) for normally distributed data. Statistical analyses were conducted using the Wilcoxon rank-sum test, Student’s t-test, or Chi-square test as appropriate. Kaplan-Meier curves were generated for both the pre-matched and matched cohorts to assess the survival of the beta blocker and non-beta blocker groups.

We examined the relationship between beta-blocker utilization and outcomes using a multivariate Cox proportional hazard model. Hazard ratios (HRs) with corresponding 95% confidence intervals (95% CI) were reported. We controlled for confounding factors, including baseline demographic characteristics and clinical parameters, as presented in Table 1. To further validate the adjustment, we performed nearest propensity score matching (PSM) on the variables listed in Table 1. Matching individuals with a caliper of 0.2, we assessed the balance between matched groups by calculating the standard mean difference (SMD). In addition, we employed inverse probability treatment weighting (IPTW), standardized mortality ratio weighting (SMRW), pairwise algorithmic (PA) (Li and Greene, 2013), and overlap weight (OW) (Li et al., 2018) models to create weighted cohorts. The individually matched and pseudo-population models demonstrated a well-balanced distribution of characteristics among groups, with a threshold of 0.1 for the standardized mean difference (SMD) to indicate imbalance.

**TABLE 1:** Baseline characteristics between groups before and after PSM.

Variables	Unmatched patients		SMD	Matched patients		SMD
	No beta-blockers	Beta-blockers		No beta-blockers	Beta-blockers	
n	615	489		364	364	
Age (years)	53.07 (16.72)	62.39 (15.37)	0.581	59.32 (15.42)	59.95 (15.94)	0.04
Male, sex, n (%)	334 (54.3)	309 (63.2)	0.181	221 (60.7)	213 (58.5)	0.045
Weight (kg)	86.46 (28.53)	86.06 (23.87)	0.015	85.26 (28.49)	85.62 (24.78)	0.014
Ethnicity, n (%)			0.122			0.039
Black	323 (52.5)	286 (58.5)		208 (57.1)	208 (57.1)	
White	36 (5.9)	23 (4.7)		17 (4.7)	20 (5.5)	
Others	256 (41.6)	180 (36.8)		139 (38.2)	136 (37.4)	
SAPS II score	45.07 (16.00)	47.41 (14.90)	0.151	47.49 (15.79)	47.68 (15.15)	0.012
SOFA score	10.42 (4.35)	10.02 (4.05)	0.096	10.37 (4.38)	10.36 (4.05)	0.002
Heart rate (bpm)	92.11 (17.48)	91.35 (18.39)	0.042	91.91 (17.81)	91.74 (18.31)	0.009
MAP (mmHg)	76.43 (9.81)	77.01 (9.56)	0.059	76.42 (9.23)	76.17 (9.53)	0.026
Respiratory rate (bpm)	22.89 (4.95)	21.38 (4.30)	0.326	21.84 (4.68)	21.97 (4.36)	0.027
Temperature (°C)	37.05 (0.88)	37.05 (0.77)	0.001	37.04 (0.84)	37.05 (0.78)	0.01
Spo2(%)	96.10 (3.52)	96.56 (2.54)	0.151	96.39 (3.00)	96.33 (2.64)	0.019
Comorbidity disease, n (%)						
Liver disease	84 (13.7)	45 (9.2)	0.14	36 (9.9)	37 (10.2)	0.009
Renal disease	76 (12.4)	96 (19.6)	0.199	62 (17.0)	62 (17.0)	<0.001
COPD	84 (13.7)	87 (17.8)	0.114	63 (17.3)	65 (17.9)	0.014
Malignancy	119 (19.3)	103 (21.1)	0.043	77 (21.2)	81 (22.3)	0.027
Sepsis	590 (95.9)	468 (95.7)	0.011	350 (96.2)	347 (95.3)	0.041
Cad	51 (8.3)	110 (22.5)	0.401	47 (12.9)	51 (14.0)	0.032
ARDS severity, n (%)			0.181			0.066
Mild	78 (12.7)	88 (18.0)		51 (14.0)	54 (14.8)	
Moderate	275 (44.7)	228 (46.6)		181 (49.7)	169 (46.4)	
Severe	262 (42.6)	173 (35.4)		132 (36.3)	141 (38.7)	
pH	7.31 (0.12)	7.31 (0.12)	0.043	7.31 (0.12)	7.31 (0.12)	0.041
PaCO_2_ (mmHg)	46.03 (15.90)	45.15 (14.32)	0.058	45.15 (13.96)	44.86 (14.84)	0.02
PaO_2_ (mmHg)	108.82 (80.12)	141.80 (107.58)	0.348	123.15 (90.26)	120.68 (88.09)	0.028
Bicarbonate	21.21 (5.74)	21.19 (5.22)	0.003	21.17 (5.74)	20.96 (5.26)	0.038
fio_2_ (%)	81.07 (22.73)	79.53 (23.43)	0.067	79.52 (22.99)	79.17 (23.52)	0.015
Mechanical power (J/min)	22.34 (9.60)	21.13 (9.52)	0.127	21.81 (9.31)	21.57 (9.85)	0.025
Driven pressure (cm H2O)	12.48 (4.43)	12.21 (3.49)	0.067	12.29 (3.61)	12.38 (3.64)	0.026
Tidal volume (ml/kg PBW)	7.83 (6.14)	7.95 (4.60)	0.022	8.10 (7.86)	7.96 (5.19)	0.022
PEEP, (cm H_2_O)	9.23 (4.07)	8.64 (4.56)	0.136	8.81 (3.88)	8.84 (4.84)	0.007
RRT, n (%)	63 (10.2)	53 (10.8)	0.019	43 (11.8)	38 (10.4)	0.044
Vasopressor use, n (%)	496 (80.7)	396 (81.0)	0.008	294 (80.8)	302 (83.0)	0.057
Glucocorticoid use, n (%)	257 (41.8)	176 (36.0)	0.119	148 (40.7)	147 (40.4)	0.006
NMBA use, n (%)	59 (9.6)	81 (16.6)	0.208	50 (13.7)	49 (13.5)	0.008

All covariates were reported as the mean (standard deviation). SOFA, sequential organ failure assessment; SAPS II, Simplified acute physiology score II; bpm, beats per minute; MAP, mean arterial blood pressure; SpO_2_, pulse oximetry; RRT, renal replacement therapy; COPD, chronic obstructive pulmonary disease; CAD, cerebrovascular disease; PaCO_2_, arterial carbon dioxide tension; PaO_2_, arterial oxygen tension; PEEP, positive end-expiratory pressure; PBW, predicted body weight; NMBA, neuromuscular blocker agent; HR, hazard ratio; CI, confidence interval.

The percentage of missing values for all participants is summarized in Supplementary Table S1. To impute the missing values, a multivariate imputation approach using chained equations was employed, assuming that the missingness was random. The analysis of the complete dataset was performed using R 4.1.2 (R Foundation) and Free Statistics version 1.7, with a significance level set at *p* < 0.05.

### 2.6 Subgroup analysis and sensitivity analyses

To examine the robustness of the findings, we performed subgroup analyses stratified by age, gender, SOFA score, SAPS II score, glucocorticoid use, NMBA use, RRT use, sepsis, and ARDS severity. Additionally, we employed E-values (Haneuse et al., 2019) to explore the potential impact of unmeasured confounding on the association between beta-blocker use and 30-day mortality. The E-value represents the magnitude of an unmeasured confounder that would be necessary to nullify the observed relationship. Moreover, to assess independent associations, we conducted sensitivity analyses focused on individuals who exclusively received beta-blockers during the first half of their ICU stay, as well as those who were administered beta-blockers within the initial 48 h of ICU admission.

## 3 Results

### 3.1 Population and baseline characteristics

**FIGURE 1:**
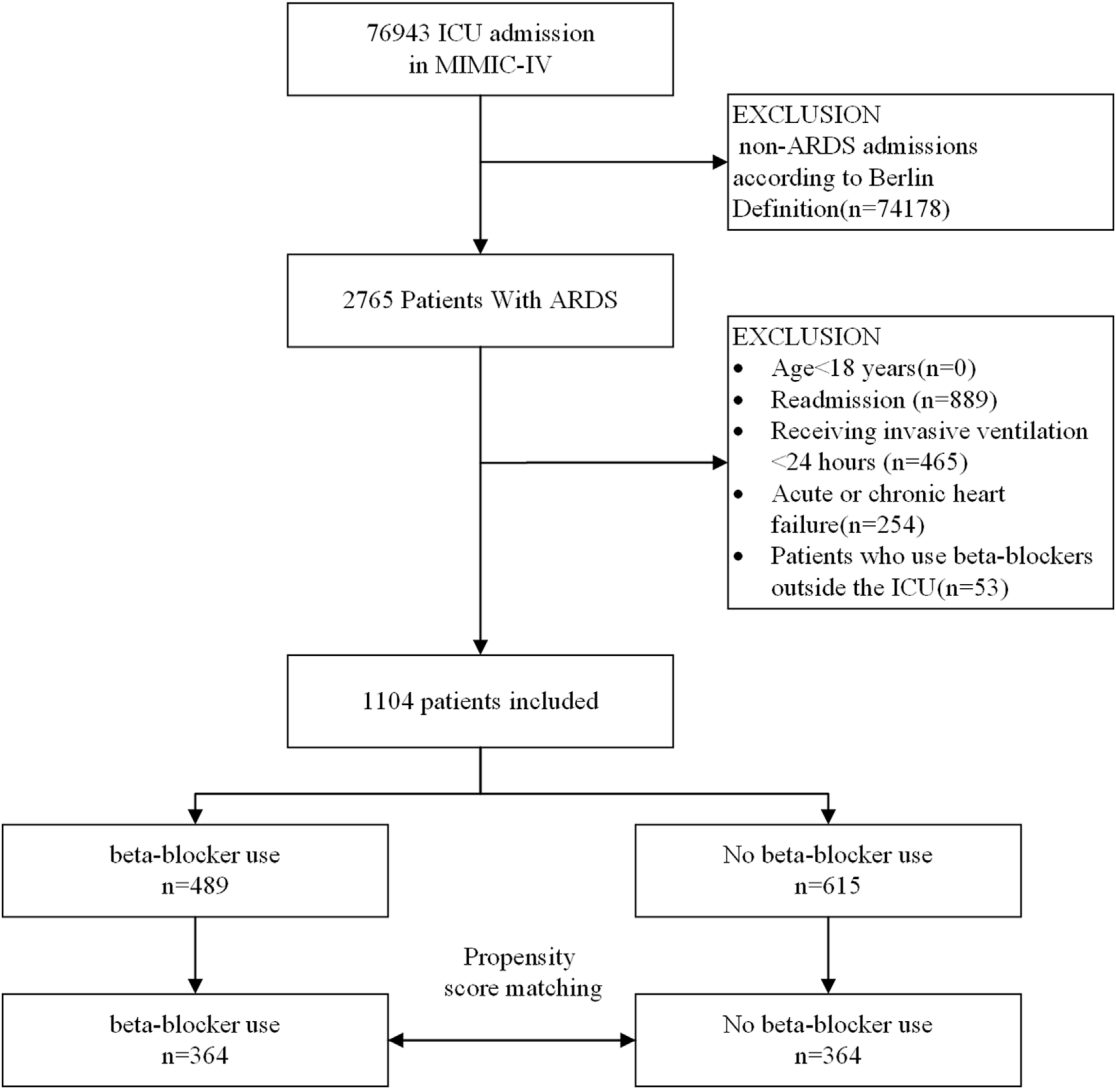
A flow chart of inclusion procedure for patients. Abbreviations: ICU, intensive care unit.

**FIGURE 2:**
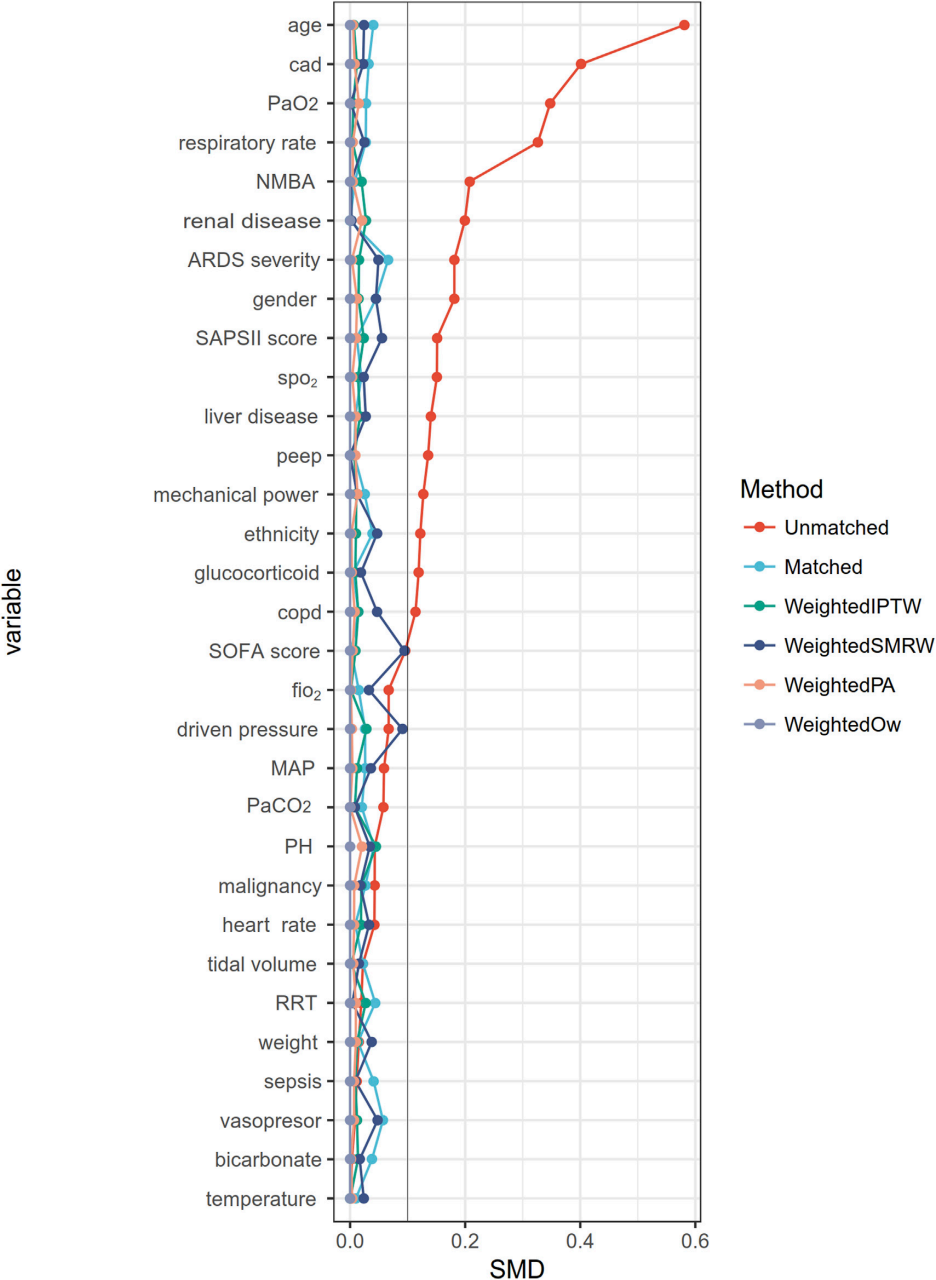
The absolute standardized mean differences for evaluating the balance of covariates between two groups. CAD, Cerebrovascular disease; NMBA, neuromuscular blocker agent; SOFA, Sequential Organ Failure Assessment; SAPS II, Simplified acute physiology score II, bpm beats per minute, MAP mean arterial blood pressure; SpO_2_, pulse oximetry; RRT, renal replacement therapy; COPD, chronic obstructive pulmonary disease; PaCO_2_, arterial carbon dioxide tension; PaO_2_, arterial oxygen tension; PEEP, positive end-expiratory pressure; PBW, predicted body weight.

A flowchart of the study process is presented in Figure 1. Following the application of exclusion criteria, a total of 1,104 patients out of 2,765 subjects identified with ARDS based on the Berlin definition were included in the analysis. Among these patients during their ICU stay, 489 (44.3%) utilized beta-blockers. The median time from ICU admission to medication initiation was 2.7 (IQR, 0.9- 6.5) days. Of these patients, 97 died in the ICU, while 243 continued drug therapy after ICU discharge, and 149 did not (Supplementary Figure S1). Among the beta-blockers, metoprolol was the most commonly prescribed, accounting for 86.1% (421 out of 489), followed by esmolol at 9.2% (45 out of 489), and labetalol at a lower prescription rate of 5.9% (27 out of 489). After propensity score matching (PSM), a group of 364 patients who received beta-blockers was paired with another group of 364 patients who did not. The patient characteristics were well-balanced between the two groups (SMD < 0.1, as shown in Table 1; Figure 2). The median age of the participants was 58.6 years, with 41.8% being female. In terms of ARDS severity, there were 54, 169, and 141 patients with mild, moderate, and severe ARDS, respectively (Table 1).

### 3.2 Primary outcome

**FIGURE 3:**
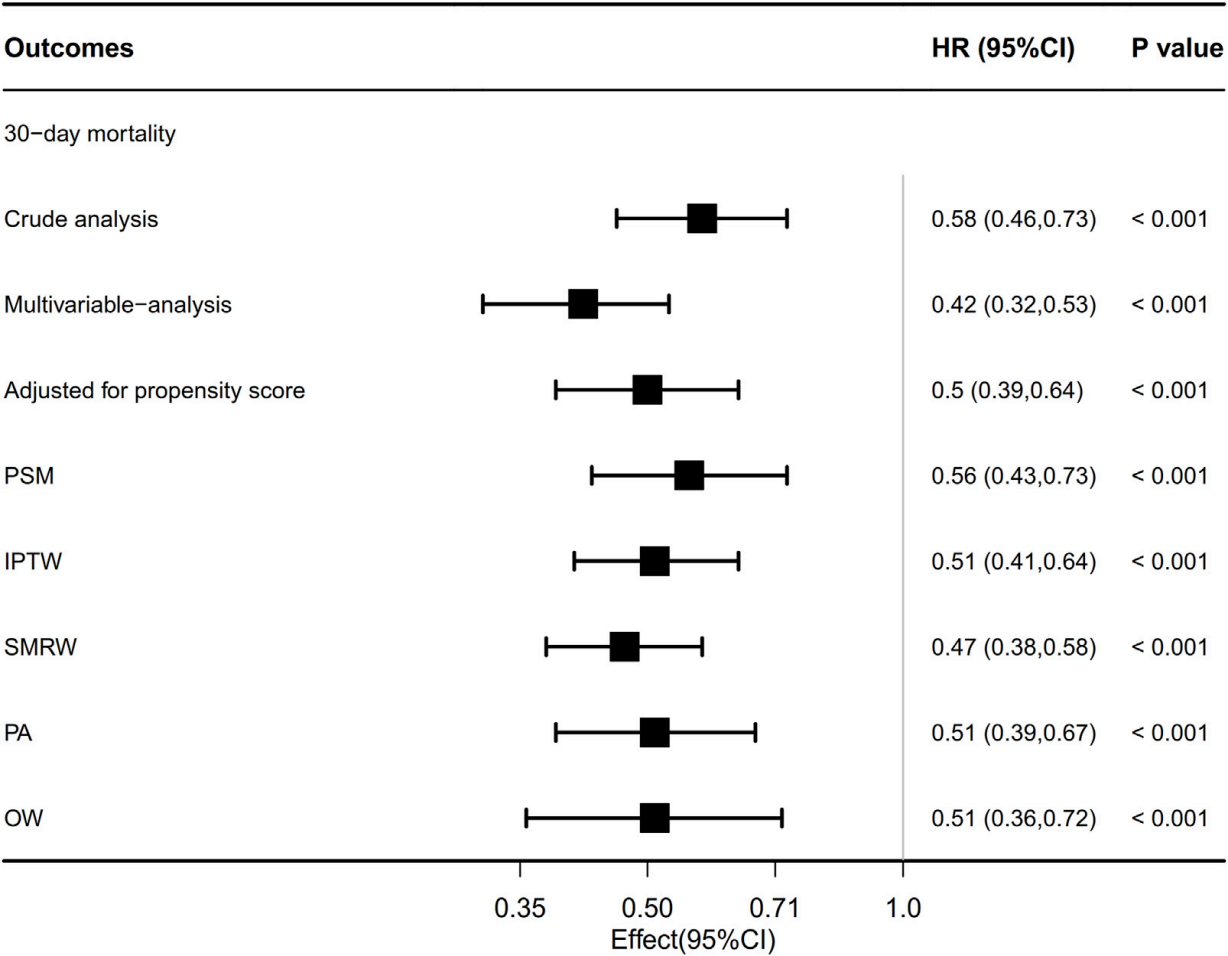
Association between beta-blocker use and ARDS patient’s outcome. Multivariable adjusted: Shown is the hazard ratio from the multivariable Cox proportional hazards model, with adjusted for all covariates in Table 1. Adjusted for propensity score: Shown is the hazard ratio from a multivariable Cox proportional-hazards model with the same strata and covariates, with additional adjustment for the propensity score; IPTW, inverse probability treatment weighting; SMRW, the standardized mortality ratio weighting. PA, pairwise algorithmic; OW, overlap weight. HR, hazard ratio; CI, confidence interval.

**FIGURE 4:**
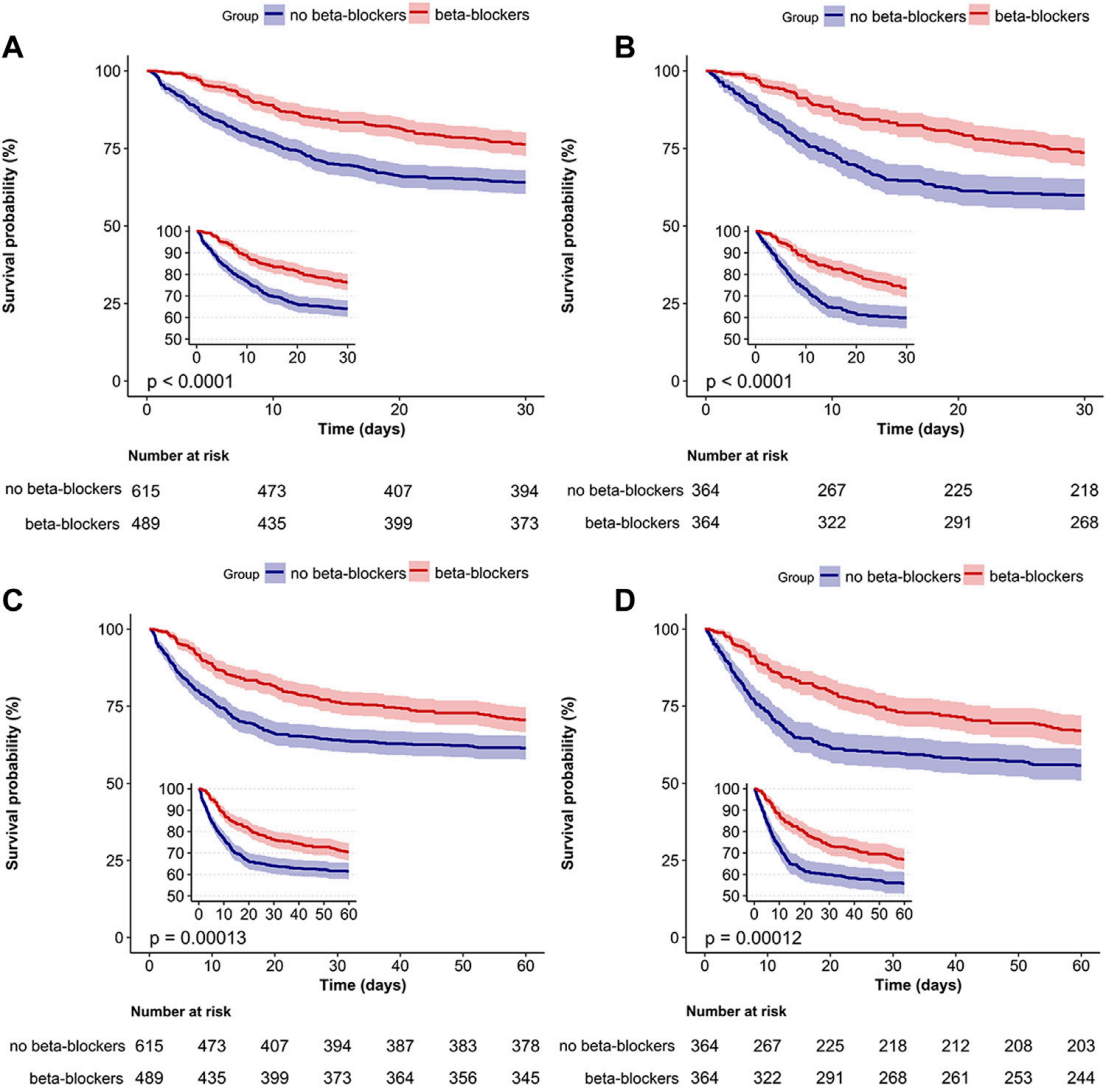
Survival analysis of beta-blockers and no beta-blockers groups. Kaplan–Meier survival curves for the 30-day **(A,B)** and 60-day **(C,D)** mortality among all patients are shown. Kaplan–Meier survival curves for pre-matched cohort **(A,C)** and matched cohort **(B,D)**.

The overall 30-day mortality rate was 30.5% (337/1104). The groups of patients who did not receive beta-blockers and those who received beta-blockers had 30-day mortality rates of 35.9% (221/615) and 23.7% (116/489), respectively (Table 2). The Kaplan-Meier curve for patients utilizing beta-blockers exhibited a significant reduction in 30-day mortality (*p* < 0.0001; Figure 4A). Univariate and multivariate Cox regression analyses, adjusted for propensity score, PSM, SMRW, PA, and OW, consistently showed a notable decrease in 30-day mortality among patients in the beta-blockers group. The HRs ranged from 0.42 to 0.58, all with *p* < 0.001 (Table 2; Figure 3; Supplementary Table S2). Consistent with the pre-matching cohort, the Kaplan-Meier survival curves indicated a lower 30-day mortality rate for patients in the beta-blockers group compared to those in the non-beta-blockers group (*p* < 0.0001; Figure 4B).

**TABLE 2:** Association between beta-blocker use and clinical outcomes in patients with ARDS.

	Overall	No beta-blockers	Beta-blockers	*p* value	HR (95% CI)	*p* value
Pre-matched cohort	*n* = 1,104	*n* = 615	*n* = 489			
30-day mortality, n (%)^a^	337 (30.5)	221 (35.9)	116 (23.7)	<0.001	0.42 (0.32–0.53)	<0.001
60-day mortality, n (%)^a^	381 (34.5)	237 (38.5)	144 (29.4)	0.002	0.49 (0.39–0.61)	<0.001
Length of ICU stay	13.2 (10.6)	11.0 (9.5)	15.9 (11.2)	<0.001	—	—
Length of hospital stay	21.0 (17.9)	17.5 (15.1)	25.5 (20.0)	<0.001	—	—
Duration of ventilation	9.7 (9.0)	8.2 (7.9)	11.7 (9.8)	<0.001	—	—
Post-matched cohort	*n* = 728	*n* = 364	*n* = 364			
30-day mortality, n (%)^b^	242 (33.2)	146 (40.1)	96 (26.4)	<0.001	0.56 (0.43–0.73)	<0.001
60-day mortality, n (%)^b^	281 (38.6)	161 (44.2)	120 (33)	0.002	0.63 (0.5–0.8)	<0.001
Length of ICU stay	13.8 (10.9)	11.3 (9.8)	16.2 (11.3)	<0.001	—	—
Length of hospital stay	21.7 (18.6)	17.7 (15.7)	25.7 (20.3)	<0.001	—	—
Duration of ventilation	10.2 (9.3)	8.4 (8.2)	12.1 (10.1)	<0.001	—	—

All covariates were reported as the mean (standard deviation).

^a^ Adjusted results obtained from multivariable Cox proportional hazards regression model that included the full cohort, with adjusted for all covariates in Table 1.

^b^ Results of univariable analysis of propensity score matched cohort. HR, hazard ratio; CI, confidence interval.

### 3.3 Propensity score matching for secondary outcomes studies

The 60-day mortality rate was 34.5% (381/1104), with rates of 38.5% (237/615) and 29.4% (144/489) observed for the groups without and with beta-blockers, respectively. After PSM, the HR for beta-blocker use was 0.63 (95% CI: 0.5–0.8, *p* < 0.001; Table 2). The Kaplan-Meier curve comparing 60-day mortality before and after PSM revealed a statistically significant difference (*p* < 0.001) in favor of beta-blocker use (Figures 4C, D). Furthermore, beta-blocker users had significantly longer lengths of stay in the intensive care unit and hospital, as well as a longer duration of ventilation, compared to non-beta-blocker users [16.2 (11.3) vs. 11.3 (9.8), *p* < 0.001; 25.7 (20.3) vs. 17.7 (15.7), *p* < 0.001; 12.1 (10.1) vs. 8.4 (8.2), *p* < 0.001; Table 2].

### 3.4 Subgroup analyses and sensitivity analysis

**FIGURE 5:**
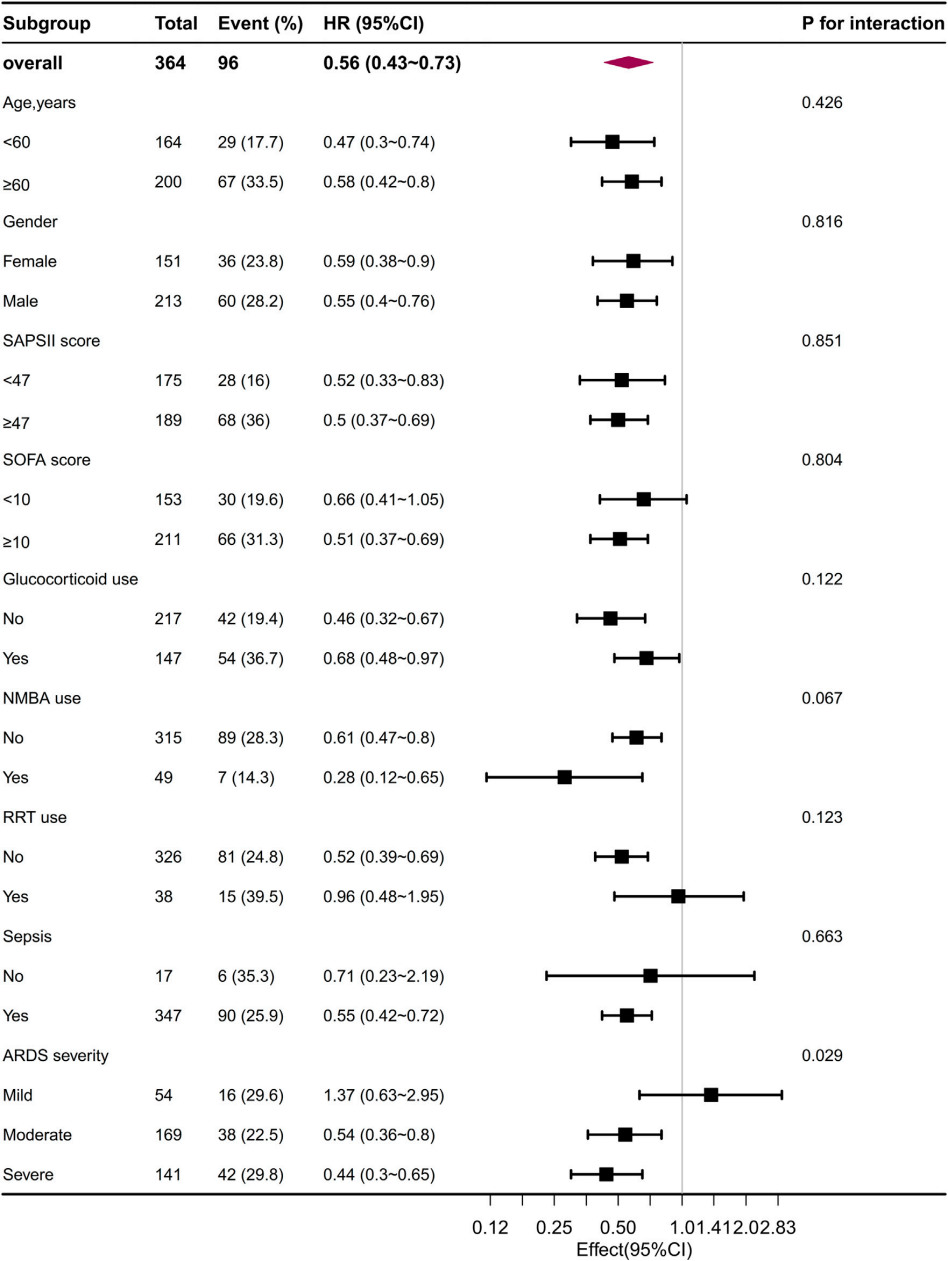
The association between beta-blocker administration and 30-day mortality in the subgroups after PSM. PSM, propensity score matching.

The subgroup analyses revealed a negative correlation between the use of beta-blockers and 30-day mortality in patients with ARDS, as depicted in Figure 5. In most subgroups, no significant interactions were observed during the subgroup analysis. However, an interaction was noted between beta-blocker therapy and the severity of ARDS after PSM, without adjusting for multiple comparisons. In patients with mild ARDS, the HR for beta-blocker use and 30-day mortality was 0.63 (0.36, 1.11). In patients with moderate ARDS, the HR was 0.65 (0.49, 0.86), and in patients with severe ARDS, the HR was 0.71 (0.59, 0.86). The *p*-value for the interaction was 0.039. The log-rank test for significance was only observed in the moderate and severe ARDS patients in both the KM curve before and after PSM (Supplementary Figure S2). The calculation of the E-values indicated that, to undermine the estimate, there should be unmeasured confounders linked to both the outcome and the exposure at a minimum of 2.97 times the measured confounders, with a mortality rate of 0.56 for 30 days after PSM (Supplementary Figure S3). After removing 106 patients who solely received beta-blockers during the second half of their stay in the ICU from the initial cohort (N = 998), our analysis centered on 383 patients who were administered beta-blockers during the first half of their ICU stay through the utilization of propensity score matching. This further affirmed the robustness of the relationship with a hazard ratio of 0.62 (95% CI = 0.47–0.82, *p* < 0.001; Supplementary Table S3; Supplementary Figure S4). Notably, even when considering patients who were given beta-blockers within 48 h of their admission to the ICU (N = 846), the association persisted strongly (HR 0.69, 95% CI 0.49–0.97, *p* = 0.033) (Supplementary Table S4; Supplementary Figure S5).

## 4 Discussion

This study established a correlation between the use of beta-blockers and a reduction in the mortality rate at both 30 and 60 days for critically ill patients with ARDS. Furthermore, the relationship between beta-blocker utilization and mortality was found to vary significantly based on the severity of ARDS. These findings have the potential to significantly impact the management of ARDS.

Critical illness is characterized by an exaggerated and dysregulated host/organ response to disease, which plays a crucial and central role in its development. Despite the distinct pathophysiological mechanisms observed in ARDS and other severe diseases, various host responses can act as a “bridge,” leading to severe manifestations with differing contents and convergences of contents. Upregulation of adrenergic stimulation is a prominent pathophysiological feature of critical diseases. An increasing number of studies are investigating the use of beta-blockers to attenuate this adrenergic response (Ackland et al., 2010). Numerous studies have demonstrated the effectiveness of beta-blockers in the treatment of various critically ill patients with conditions such as sepsis, prior to major surgical procedures, burns, cardiac arrest, and traumatic brain injury. These studies have significantly contributed to enhancing our understanding of the benefits provided by beta-blockers in these specific clinical scenarios (Herndon et al., 2001; van der Jagt and Miranda, 2012; Morelli et al., 2013; Chacko and Gopal, 2015; Lee et al., 2016). The BASEL II ICU study conducted by Noveanu et al. demonstrated a potential reduction in mortality among individuals with acute respiratory failure admitted to the ICU who had been taking oral beta-1 blockers prior to admission (Noveanu et al., 2010). However, this study solely focuses on beta-blocker utilization before hospital admission and upon discharge. Due to the absence of previous studies focusing specifically on the ARDS population, limited sample size, and varied baseline characteristics, potential bias may have arisen, and the impact of beta-blocker use during ICU stay on survival outcomes among ARDS patients remains unclear. To address this, our study employed PSM to mitigate selection bias and ensure baseline variable equilibrium across different groups. We found that the use of beta-blockers was associated with reduced mortality, providing evidence supporting the potential benefits of beta-blockers in improving prognosis for patients with ARDS. Furthermore, these findings highlight the pleiotropic effects of beta-blockers in critically ill patients.

Experimental studies have demonstrated the protective effects of beta-blockers on lung function. In a rat model of lipopolysaccharide-induced sepsis, Hagiwara et al. discovered that landiolol decreased the wet-to-dry lung ratio, indicating a reduction in lung edema (Hagiwara et al., 2009). Similarly, Piacentini et al. (2008) found that Esmolol improved pulmonary flow and significantly reduced lung congestion, edema, areas of inflammation, bleeding, and overall lung injury in septic animals that were spontaneously ventilating (Piacentini et al., 2008). Moreover, in pigs with endotoxic shock, the administration of beta-blockers did not significantly affect cardiac output, but led to an increase in the PaO_2_/FiO_2_ ratio after 3 h, indicating improved oxygenation (Aboab et al., 2011). Based on these findings, it is plausible to hypothesize that reducing pulmonary vascular flow through beta-blockers may mitigate lung damage.

Saifi et al. (2021) conducted an analysis of older patients with Coronavirus disease 2019 (COVID-19), with the majority (97.1%) experiencing respiratory failure. Their findings revealed a statistically significant association between beta-blocker therapy and a higher survival rate compared to the non-treated group (Saifi et al., 2021). In a case-control study of 20 COVID-19 patients with ARDS, Clemente-Moragón et al. (2021) demonstrated that metoprolol administration improved oxygenation and reduced lung inflammation. The researchers proposed that this beneficial effect of beta-blockers was achieved by modulating neutrophil function and preventing excessive inflammation. Additionally, the infusion of metoprolol in critically ill COVID-19 patients could positively impact oxygenation through hemodynamic changes (Clemente-Moragón et al., 2021). This effect may be attributed to a reduction in intrapulmonary shunting by lowering cardiac output and consequently improving ventilation-perfusion ratios (de Roquetaillade et al., 2022). A similar mechanism may explain why beta-blocker usage improves oxygenation in patients receiving veno-venous extracorporeal membrane oxygenation (V-V ECMO) (Bunge et al., 2019). Among patients with hyperdynamic circulation, beta-blockers have been shown to potentially decrease the amount of unoxygenated blood passing through the extracorporeal membrane and decrease intrapulmonary shunts. This may also provide some cardioprotective effects (Lynch et al., 1979). The research discussed in this paper focused specifically on COVID-19 patients with ARDS. It should be noted that critically ill patients can develop ARDS due to various factors, leading to diverse symptoms and comorbidities. In the MIMIC-IV database, all patients with ARDS were included regardless of the underlying cause, symptomatology, other medical conditions, or severity. The results of this study suggested that beta-blockers may have favorable outcomes in the overall ARDS population rather than a specific subgroup. However, it is important to mention that we did not examine changes in cardiac output and oxygenation, making it difficult to confirm whether the observed association is attributed to improved oxygenation through the reduction of intrapulmonary shunting with beta-blockers.

The precise mechanism by which beta-blockers exert their effects in patients with ARDS is not fully understood. However, several preclinical studies have provided valuable insights into the pleiotropic effects of beta-blockers. For example, a study in rats with lipopolysaccharide sepsis demonstrated that landiolol can inhibit lung injury and reduce the expression of high mobility group protein b1 (HMGB-1), a protein associated with lung injury (Hagiwara et al., 2009). Similarly, carvedilol has been found to suppress the activation of the nucleotide-binding oligomerization domain-like receptor containing pyrin domain 3 (NLRP3) inflammasome, leading to a reduction in pyroptosis and inflammatory cytokine levels in alveolar macrophages (AMs), ultimately resulting in decreased lung injury (Wong et al., 2018). Furthermore, certain beta-blockers, such as nebivolol, have been shown to enhance the bioavailability of nitric oxide and improve endothelial function in ARDS (Vassiliou et al., 2020; Kumar et al., 2021). These studies illustrate distinct mechanisms of action for beta-blockers in ARDS and emphasize the need for further investigation in this area.

Our study revealed an intriguing finding regarding beta-blockers, which displayed an extended duration of hospitalization and stay in the ICU, despite lower mortality rates. This observation aligns with previous research conducted on patients with traumatic brain injury or sepsis (Ley et al., 2018; Ge et al., 2023). However, we cannot definitively conclude whether this disparity is due to a trade-off between mortality and length of stay in the ICU/hospital. It is plausible that early mortality might have reduced the average duration of stay in the beta-blocker non-user group, necessitating further investigation. Importantly, sub-analyses examining different ARDS severities revealed a significant interaction. Specifically, the beneficial effects of beta-blockers on 30-day survival were only evident in patients with moderate or severe ARDS. In contrast, patients with mild ARDS might not exhibit the same magnitude of dysregulated host/organ response as those with moderate or severe ARDS, thereby potentially limiting the benefits of beta-blocker therapy at this level of disease severity.

This study has multiple noteworthy limitations. Firstly, like all retrospective analyses, there is a possibility of residual confounding factors. Nonetheless, we diligently endeavored to control for confounders and successfully achieved optimal balance among the PSM, IPTW, SMRW, PA, and OW groups. Secondly, it is important to consider that different treatment strategies, such as prone position ventilation and extracorporeal membrane oxygenation (ECMO) utilization, may influence patient outcomes. Thirdly, although a range was provided in the descriptive statistics, we did not specify the exact dosage and timing of beta-blocker administration in this study. Additionally, the impact of different types of beta-blockers (long-acting vs. short-acting) on prognosis was not deeply explored. Fourthly, due to the limited availability of hemodynamic measures (e.g., cardiac output) for ARDS patients in the database, we could not accurately assess the precise hemodynamic status associated with beta-blocker usage. Fifthly, the heterogeneity of ARDS may impact the treatment effect in distinct phenotypes (Beitler et al., 2022). Thus, our findings cannot be universally applied to outcomes across all phenotypes, necessitating future studies to address specific phenotypes. Finally, given that the data analyzed was derived from an observational database, further randomized trials are warranted to validate our findings.

## 5 Conclusion

Based on findings from this cohort study, the administration of beta-blockers demonstrated an association with reduced mortality in patients with ARDS. These results suggest that beta-blockers hold promise as a potential adjunctive treatment approach for ARDS patients in the ICU. However, it is important to acknowledge the retrospective nature of this study and the need for further investigation to validate these findings.

## Data Availability

Publicly available datasets were analyzed in this study. This data can be found here: https://physionet.org/content/mimiciv/2.0/.
