# Supplementary material for: Effects of beta-blockers use on mortality of patients with acute respiratory distress syndrome: a retrospective cohort study
Source: Front Physiol. 2024 Jan 19;15:1332571. doi: 10.3389/fphys.2024.1332571 (PMC10834676; doi:10.3389/fphys.2024.1332571)
Supplement: Supplementary file 1 [file Table1.docx]

**Supplementary Material**

| **Table S1** Percentage of missing data in the variables in the interest | | |  |
| --- | --- | --- | --- |
| Variable | Missing number | Missing percentage (%) |  |
| Age(years) | 0 | 0 |  |
| gender | 0 | 0 |  |
| Weight | 18 | 1.63 |  |
| Ethnicity | 0 | 0 |  |
| SAPS II score | 0 | 0 |  |
| SOFA score | 0 | 0 |  |
| Heart rate | 0 | 0 |  |
| MAP | 0 | 0 |  |
| Respiratory rate | 0 | 0 |  |
| temperature | 50 | 4.53 |  |
| spo2 | 0 | 0 |  |
| Liver disease | 0 | 0 |  |
| Renal disease | 0 | 0 |  |
| CAD | 0 | 0 |  |
| COPD | 0 | 0 |  |
| malignancy | 0 | 0 |  |
| sepsis | 0 | 0 |  |
| ARDS severity | 0 | 0 |  |
| PH | 40 | 3.62 |  |
| PaCO2 | 45 | 4.08 |  |
| PaO2 | 45 | 4.08 |  |
| fio2 | 2 | 0.18 |  |
| bicarbonate | 0 | 0 |  |
| mechanical power | 95 | 8.61 |  |
| Tidal volume (ml/kg PBW) | 152 | 13.77 |  |
| PEEP | 0 | 0 |  |
| Driven pressure | 94 | 8.51 |  |
| glucocorticoid use | 0 | 0 |  |
| NMBA use | 0 | 0 |  |
| RRT | 0 | 0 |  |
| Vasopressor use | 0 | 0 |  |
| beta-blocker use | 0 | 0 |  |
| 30-day mortality | 0 | 0 |  |
| 60-day mortality | 0 | 0 |  |
| Length of hospital stay | 0 | 0 |  |
| Length of ICU stay | 0 | 0 |  |
| Duration of ventilation | 0 | 0 |  |
| SOFA: Sequential Organ Failure Assessment, SAPSII, Simpliﬁed acute physiology score II, bpm: beats per minute, MAP: mean arterial blood pressure, SpO2 pulse oximetry, RRT: renal replacement therapy, COPD: chronic obstructive pulmonary disease, CAD: Cerebrovascular disease, PaCO2 arterial carbon dioxide tension, PaO2: arterial oxygen tension, PEEP: positive end-expiratory pressure, PBW: predicted body weight, NMBA: neuromuscular blocker agent, HR: hazard ratio; CI: confidence interval. | | |  |
|  |  |  |  |
|  |  |  |  |
|  |  |  |  |
|  |  |  |  |
|  |  |  |  |

| **Table S2.** Multivariate cox regression analysis for risk of 30-day mortality | | | |  |
| --- | --- | --- | --- | --- |
| Variable | HR | 95%CI | P_value |  |
|  |  |  |  |  |
| beta-blockers use | 0.42 | 0.32~0.53 | <0.001 |  |
| age | 1.02 | 1.02~1.03 | <0.001 |  |
| gender | 1.38 | 1.08~1.77 | 0.011 |  |
| weight | 0.99 | 0.99~1 | 0.025 |  |
| Ethnicity |  |  |  |  |
| White | 0.72 | 0.4~1.3 | 0.273 |  |
| Others | 1.51 | 1.2~1.91 | 0.001 |  |
| RRT | 0.87 | 0.61~1.23 | 0.421 |  |
| Vasopressor use | 1.7 | 1.13~2.56 | 0.01 |  |
| SAPS II score | 1.02 | 1.01~1.03 | <0.001 |  |
| SOFA score | 1.03 | 0.99~1.07 | 0.182 |  |
| Heart rate | 1.01 | 1.01~1.02 | <0.001 |  |
| MAP | 0.99 | 0.98~1.01 | 0.218 |  |
| Respiratory rate | 1.04 | 1.01~1.07 | 0.003 |  |
| temperature | 0.56 | 0.48~0.64 | <0.001 |  |
| spo2 | 0.96 | 0.93~1 | 0.038 |  |
| Liver disease | 1.64 | 1.21~2.23 | 0.001 |  |
| Renal disease | 1.28 | 0.95~1.72 | 0.108 |  |
| COPD | 0.79 | 0.57~1.09 | 0.153 |  |
| malignancy | 0.95 | 0.72~1.26 | 0.735 |  |
| sepsis | 0.5 | 0.3~0.84 | 0.008 |  |
| CAD | 1.46 | 1.07~1.99 | 0.017 |  |
| PH | 2.18 | 0.39~12.18 | 0.373 |  |
| PaCO2 | 1 | 0.99~1.01 | 0.92 |  |
| PaO2 | 1 | 1~1 | 0.758 |  |
| bicarbonate | 1.02 | 0.98~1.05 | 0.395 |  |
| ARDS severity |  |  |  |  |
| Moderate | 0.82 | 0.57~1.19 | 0.298 |  |
| Severe | 0.82 | 0.53~1.25 | 0.349 |  |
| fio2 | 1 | (0.99~1 | 0.284 |  |
| mechanical power | 1 | 0.99~1.01 | 0.972 |  |
| Driven pressure | 1.01 | 0.98~1.04 | 0.449 |  |
| Tidal volume | 1.01 | 0.99~1.02 | 0.306 |  |
| PEEP | 1.03 | 0.99~1.06 | 0.11 |  |
| glucocorticoid use | 1.43 | 1.13~1.8 | 0.002 |  |
| NMBA use | 0.72 | 0.49~1.05 | 0.09 |  |
| SOFA: Sequential Organ Failure Assessment, SAPSII, Simpliﬁed acute physiology score II, bpm: beats per minute, MAP: mean arterial blood pressure, SpO2 pulse oximetry, RRT: renal replacement therapy, COPD: chronic obstructive pulmonary disease, CAD: Cerebrovascular disease PaCO2: arterial carbon dioxide tension, PaO2: arterial oxygen tension, PEEP: positive end-expiratory pressure, PBW: predicted body weight, NMBA: neuromuscular blocker agent,HR: hazard ratio; CI: confidence interval. | | | |  |
|  |  |  |  |  |
|  |  |  |  |  |
|  |  |  |  |  |
|  |  |  |  |  |
|  |  |  |  |  |

| **Table S3.** Sensitivity analysis of beta-blockers use within only during the first half of the ICU stay | | |  |
| --- | --- | --- | --- |
| Analysis | 30-day mortality (%) | P-value |  |
| No. of events/no. of patients at risk (%) |  |  |  |
| No beta-blocker use | 221/615(35.9) |  |  |
| beta-blocker use | 95/383 (24.8) |  |  |
| Crude analysis — hazard ratio (95% CI) | 0.61 (0.48,0.77) | < 0.001 |  |
| Multivariable analysis — hazard ratio (95% CI) | 0.43 (0.33,0.56) | < 0.001 |  |
| Adjusted for propensity score | 0.54 (0.41,0.7) | < 0.001 |  |
| With matching | 0.62 (0.47,0.82) | < 0.001 |  |
| With IPTW | 0.54 (0.42,0.69) | < 0.001 |  |
| With SMRW | 0.52 (0.41,0.66) | < 0.001 |  |
| With PA | 0.55 (0.41,0.73) | < 0.001 |  |
| With OW | 0.54 (0.38,0.78) | 0.001 |  |
| Multivariable adjusted: Shown is the hazard ratio from the multivariable Cox proportional hazards model, with adjusted for all covariates in Table 1. Adjusted for propensity score: Shown is the hazard ratio from a multivariable Cox proportional-hazards model with the same strata and covariates, with additional adjustment for the propensity score; IPTW, inverse probability treatment weighting; SMRW, the standardized mortality ratio weighting.PA, Pairwise algorithmic; OW, overlap weight. CI, confidence interval. | | |  |
|  |  |  |  |
|  |  |  |  |
|  |  |  |  |
|  |  |  |  |
|  |  |  |  |

| **Table S4.** Sensitivity analysis of beta-blockers use only within 48h after ICU admission | | |  |  |
| --- | --- | --- | --- | --- |
| Analysis | 30-day mortality (%) | P-value | |  |
| No. of events/no. of patients at risk (%) |  |  | |  |
| No beta-blocker use | 219/611 (35.8) |  | |  |
| beta-blocker use | 66/235(28.1) |  | |  |
| Crude analysis — hazard ratio (95% CI) | 0.71 (0.54,0.94) | 0.017 | |  |
| Multivariable analysis — hazard ratio (95% CI) | 0.54 (0.39,0.74) | < 0.001 | |  |
| Adjusted for propensity score | 0.65 (0.47,0.88) | 0.006 | |  |
| With matching | 0.69 (0.49,0.97) | 0.033 | |  |
| With IPTW | 0.74 (0.56,0.97) | 0.031 | |  |
| With SMRW | 0.62 (0.48,0.82) | < 0.001 | |  |
| With PA | 0.63 (0.44,0.89) | 0.01 | |  |
| With OW | 0.66 (0.43,1) | 0.05 | |  |
| Multivariable adjusted: Shown is the hazard ratio from the multivariable Cox proportional hazards model, with adjusted for all covariates in Table 1. Adjusted for propensity score: Shown is the hazard ratio from a multivariable Cox proportional-hazards model with the same strata and covariates, with additional adjustment for the propensity score; IPTW, inverse probability treatment weighting; SMRW, the standardized mortality ratio weighting.PA, Pairwise algorithmic; OW, overlap weight. CI, confidence interval. | | |  |  |
|  |  |  |  | |
|  |  |  |  | |
|  |  |  |  | |
|  |  |  |  | |
|  |  |  |  | |

**
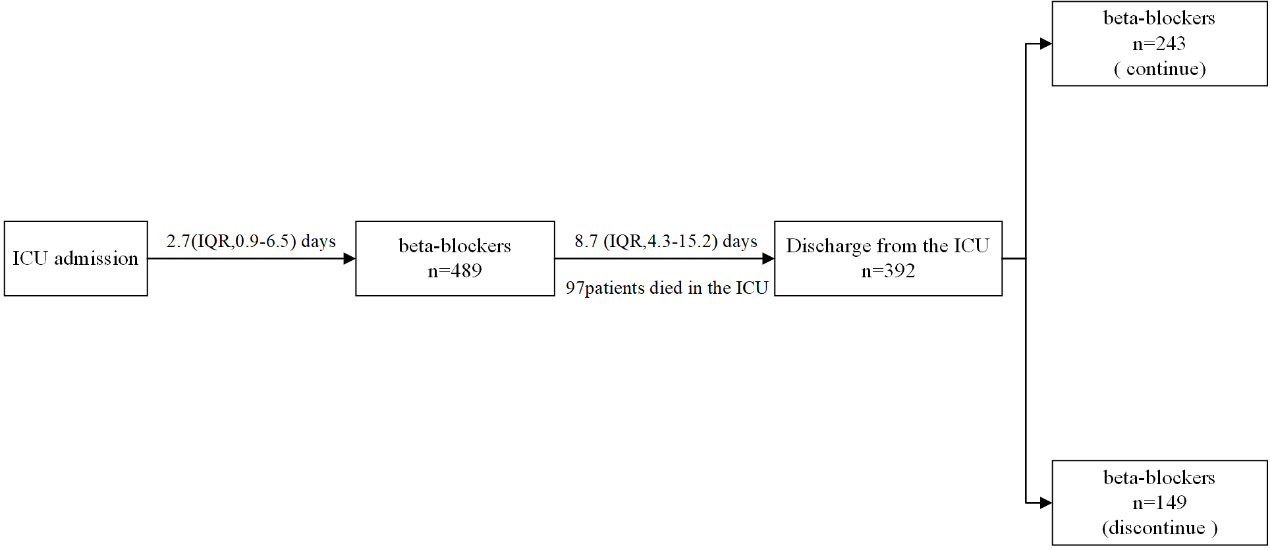
**

Figure S1. Progress of beta-blocker therapy during course of hospitalization. Data are presented as median (IQR). ICU, intensive care unit; IQR, interquartile range.

**
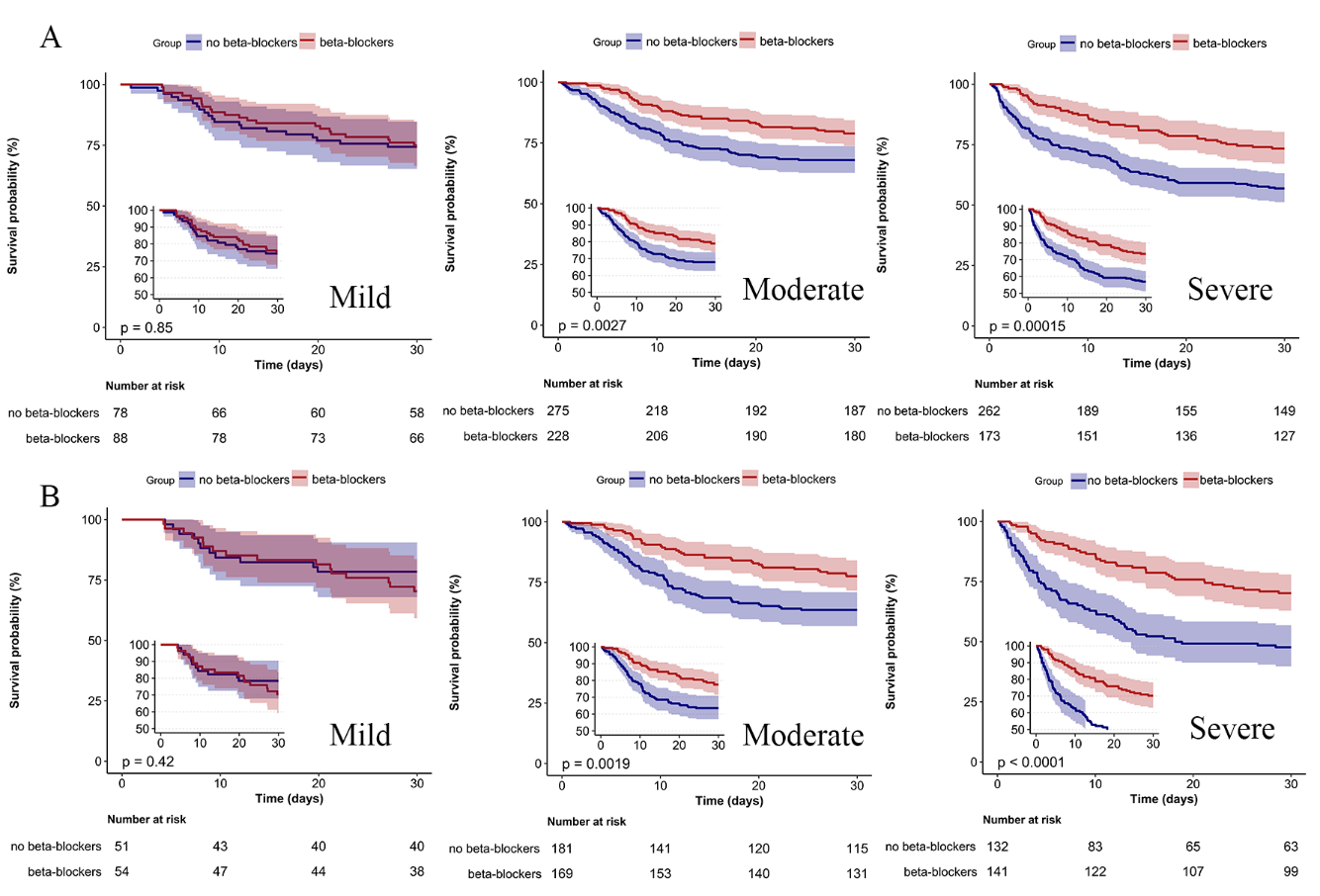
**

Figure S2. Survival curve of patients with different severities of ARDS. (A) Survival curve of patients with different severities of ARDS before PSM. (B) Survival curve of patients with different severities of ARDS after PSM

**
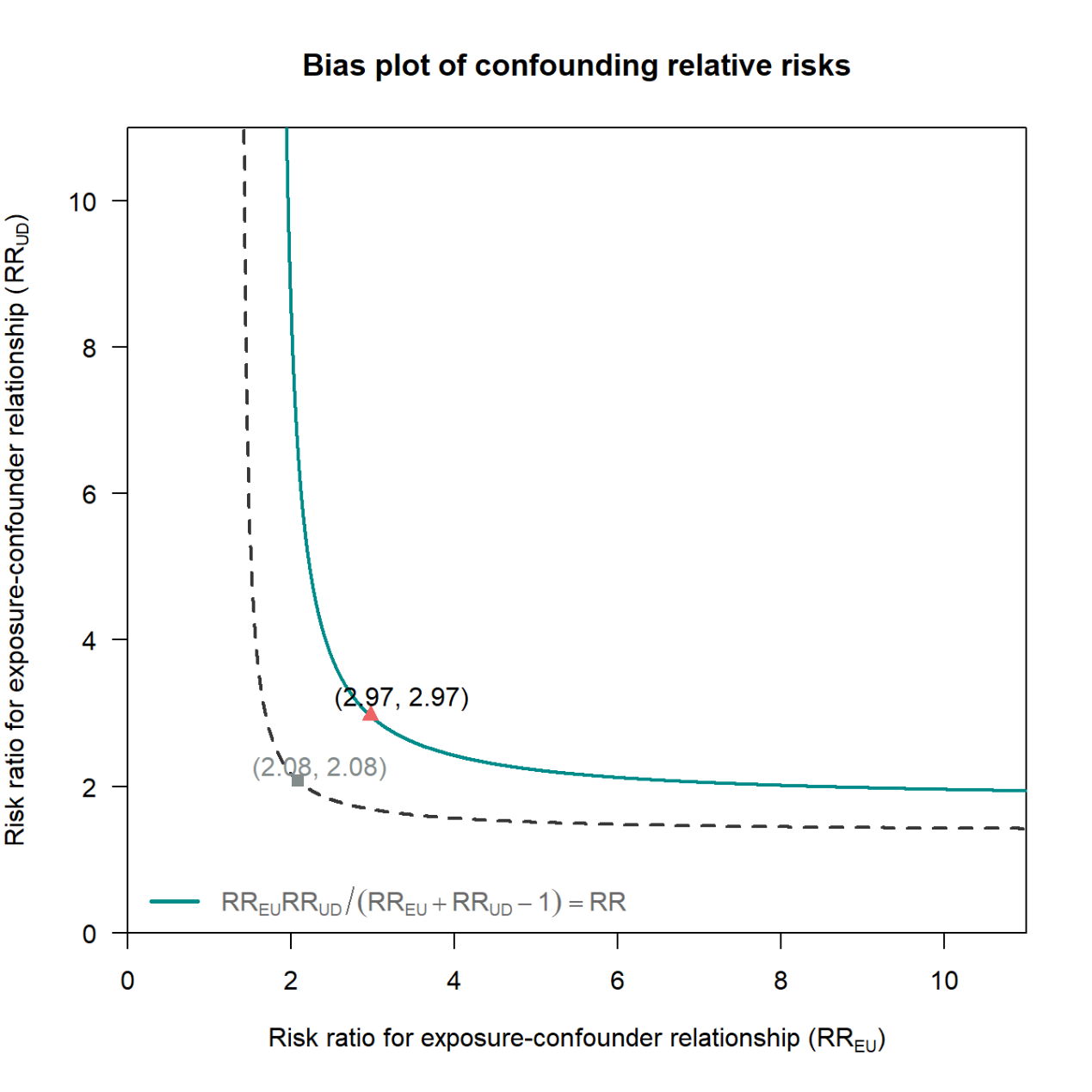
**

Figure S3. Estimation of E-values of association between beta-blockers and mortality

**
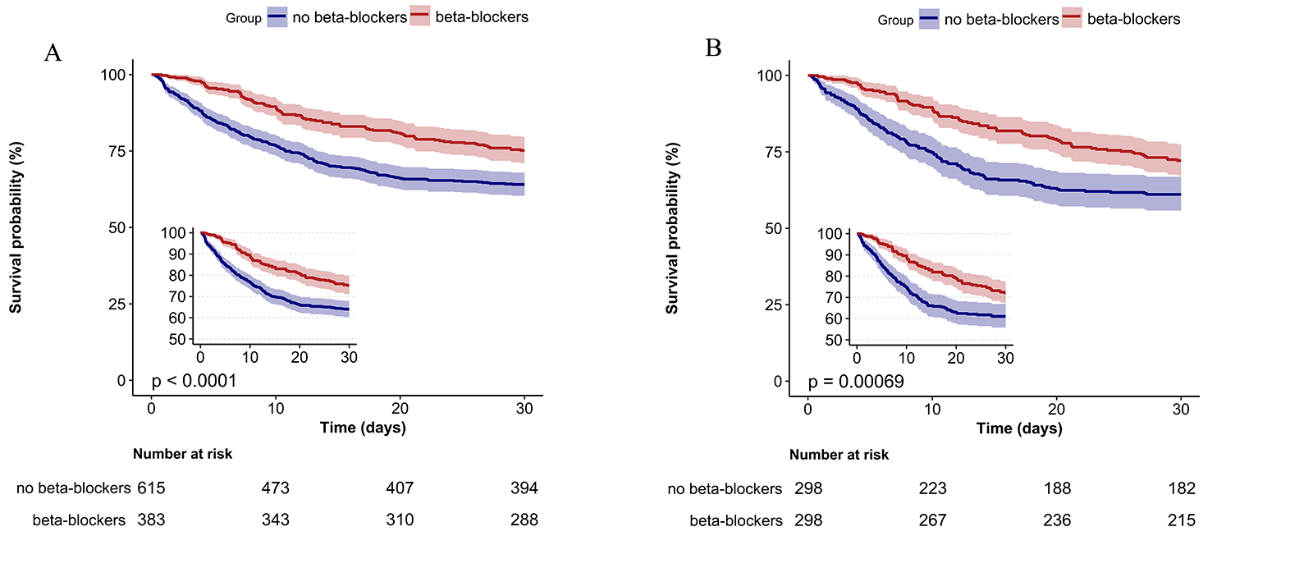
**

**Figure S4.** Survival analysis of beta-blockers and no beta-blockers groups within only during the first half of the ICU stay. Kaplan–Meier survival curves for pre-matched cohort (A) and matched cohort (B).

**
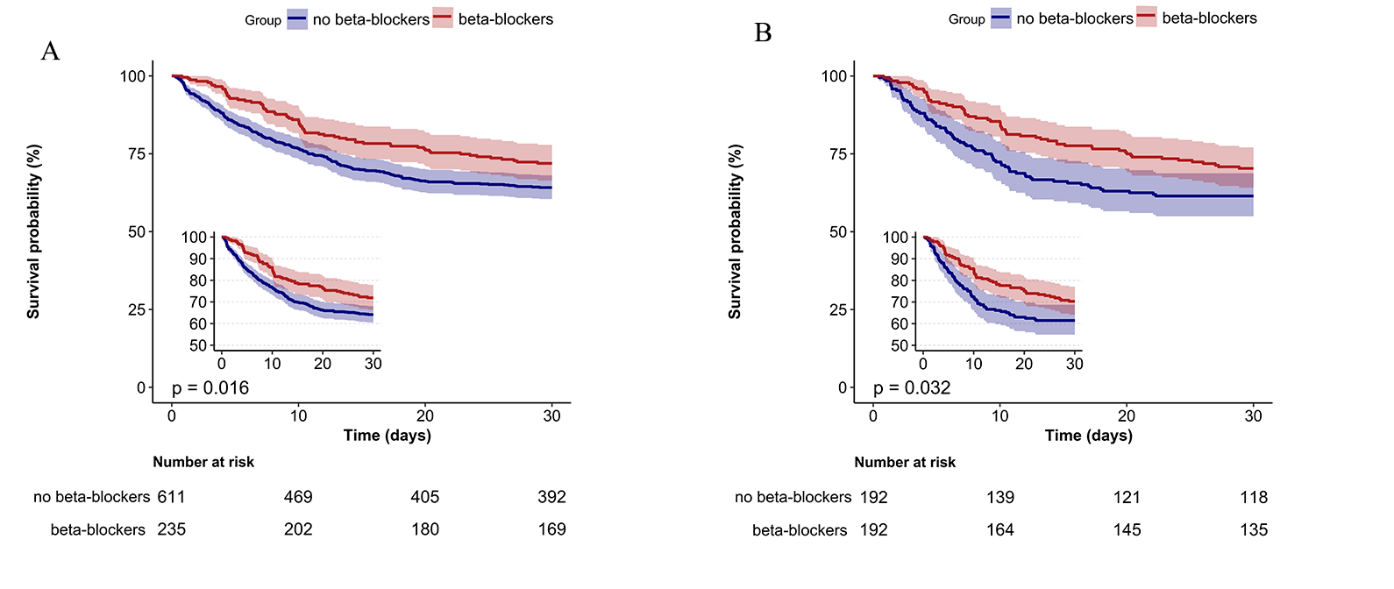
**

**Figure S5.** Survival analysis of beta-blockers and no beta blockers groups only within 48h after ICU admission. Kaplan–Meier survival curves for pre-matched cohort (A) and matched cohort (B).
